# Genetic structure of sugar kelp in the St. Lawrence Estuary and Gulf (Québec, Canada)

**DOI:** 10.1111/jpy.70141

**Published:** 2026-02-28

**Authors:** Marie Treillefort, Sabrina Le Cam, Myriam Valero, Stéphane Mauger, Paolo Ruggeri, Flora Salvo, Isabelle Gendron‐Lemieux, Tamara Provencher, Rénald Belley, France Dufresne

**Affiliations:** ^1^ Département de Biologie Université du Québec à Rimouski Rimouski Quebec Canada; ^2^ CNRS, PUCCH, UACH, UMI 3614, Evolutionary Biology and Evolution of Algae Station Biologique de Roscoff, Sorbonne Université Roscoff France; ^3^ CNRS, UMR 7144, Dispersal Speciation and Evolution of Marine Species, Laboratory Adaptation et Diversité en Milieu Marin (AD2M) Station Biologique de Roscoff, Sorbonne Université Roscoff France; ^4^ Merinov Gaspé Quebec Canada; ^5^ École des pêches et de L'aquaculture du Québec Cégep de la Gaspésie et des Îles Grande‐Rivière Quebec Canada; ^6^ Fisheries and Oceans Canada Maurice Lamontagne Institute Mont‐Joli Quebec Canada; ^7^ Present address: CNRS, UMR7266, Littoral Environnement et Sociétés La Rochelle Université La Rochelle France; ^8^ Present address: Xelect Ltd St Andrews UK

**Keywords:** DartSeq, genomic markers, microsatellite markers, population genetics, *Saccharina latissima*, St. Lawrence Estuary and Gulf

## Abstract

The sugar kelp, *Saccharina latissima*, is cultivated at low scale in Quebec, Canada, and current practice involves seeding meiospores or gametophyte stocks onto spools carrying twine and transferring these to a seaweed farm site. As the stocks can originate from locations spanning several hundreds of kilometers from the farm sites, such practices could involve genetic contamination and disrupt local adaptations. Assessing genetic structure can inform us of the potential risks associated with these practices. Here, we characterized the genetic diversity and structure of *S. latissima* from locations in the St. Lawrence Estuary and Gulf at both microsatellite loci (308 sporophytes at 22 loci in 16 sites) and genomic markers (228 sporophytes at 6578 single nucleotide polymorphisms, or SNPs, in 13 sites). Several populations had low heterozygosity values and significant *F*_IS_ values at microsatellite loci. No genetic structure was observed among populations with microsatellite loci, but strong genetic structuring was observed with the genomic data. Population structure followed a geographic pattern and was congruent with major currents. Individuals from the wild population in the vicinity of the farm site were genetically distinct from the sporophytes on the growing lines that belong to a genetically distinct group. There was no significant genetic differentiation between wild individuals living in proximity to the farm site and another wild population of the same area. Hence, aquaculture practices have not resulted in changes in the genetic composition of the wild population on a large scale. Our results are important to guide future conservation efforts and the seaweed farming industry.

**Abbreviations**
AMOVA: analysis of molecular variance
ANOVA: analysis of variance
AR: allelic richness
BON: Bonaventure
BONDC: sporophytes from Bonaventure on growing lines
CAD: Cap Desrosiers
CAO: Cap‐aux‐Os
CASCA: Cascapedia
COL: Colbourne
COR: Cormorandière
dNTP: deoxyribonucleotide
EST: expressed‐sequence‐tag
*F*_IS_
: inbreeding coefficient
GRA: Grande‐Rivière
*H*e: expected heterozygosity
*H*o: observed heterozygosity
IV: Ile verte
LUD: Baie St‐Ludger
MgCl2: magnesium chloride
NEW: in the Baie‐des‐Chaleurs: Newport
NOR: Sept‐Iles
PAS: Paspébiac
PCA: principal component analysis
PCR: polymerase chain reaction
Purmer algaculture site: PUR: in the Gulf along the North Shore: Ile Grosse Boule
ROO: around the Iles‐de‐la‐Madeleine: Rochers‐aux‐Oiseaux
SAM: along the Gaspé Peninsula: Sainte‐Anne‐des‐Monts
sNMF: sparse non‐negative matrix factorization
SNP: single nucleotide polymorphisms
TAD: Tadoussac

## INTRODUCTION

Kelp farming has long been carried out in coastal Asia and is expanding in Europe, South America, and North America (Augyte et al., 2017; Bjerregaard et al., 2016; Breton et al., 2018; Campbell et al., 2019; Goecke et al., 2020; Grebe et al., 2019; Hwang et al., 2019; Kim et al., 2019; Tamigneaux & Johnson, 2016). Kelp is usually harvested when mature in the wild and then grown on lines and harvested for use in many industries (pharmaceutical and biomedical products, cosmetics, feed supplements, or biofuels; Bartsch et al., 2008; Forbord et al., 2012; Jahan et al., 2017; Marinho et al., 2015). *Saccharina latissima* is currently one of the main cultivated species in Europe and North America (Breton et al., 2018; Tamigneaux & Johnson, 2016). The increased popularity of this species for seaweed farming has raised consideration of developing best practices, including farming without perturbing wild populations.

In the St. Lawrence Gulf, *Saccharina latissima* has been cultivated experimentally and at low scale since 2006 (Tamigneaux & Johnson, 2016). This cultivation involves collecting mature sori from wild individuals and seeding young sporophytes on farming lines until sufficient growth is reached to harvest. Seedlings are then transferred onto lines at sites that can be located several hundreds of kilometers away from their original location. Even though sporophytes are usually harvested before developing fertile meiospores, there is a risk of genetic contamination if sporophytes are detached from the culture lines and disperse into nearby wild populations. The risk of genetic contamination depends on the extent of genetic differentiation between the population on the cultivation lines and the wild populations near the algaculture sites. Addressing the genetic diversity and genetic structure of this important founding species in the St. Lawrence Estuary and Gulf is an important step toward efficient management.

Many studies have examined the genetic structure of the sugar kelp, *Saccharina latissima*, using different types of molecular markers reflecting different time scales (Breton et al., 2018; Guzinski et al., 2016; Nielsen et al., 2016; Neiva et al., 2018). Deep biogeographic history has been revealed with mitochondrial DNA (Neiva et al., 2018). Three phylogroups corresponding to (1) NE (British Columbia) Pacific, Greenland, and Hudson Bay; (2) NE Atlantic; and (3) NW Atlantic were characterized (Neiva et al., 2018). Colonization from the NE Pacific into the Arctic and the NE Atlantic is thought to have occurred 5.5 million years ago (Luttikhuizen et al., 2018). Pleistocene glaciations have compressed NE Atlantic populations to the south and isolated NE and NW Atlantic populations (Neiva et al., 2018). At the scale of the eastern Atlantic region, northern (Norway, Sweden, Denmark) and southern (France) populations formed distinct clusters at 25 microsatellite loci, with an average *F*_ST_ of 0.36 (Guzinski et al., 2016). This strong differentiation between northern and southern Europe was refined recently based on sampling from of 24 sites, which confirmed the occurrence of two refugia, one in Southern Europe and the other at high latitude further north than northern Norway (Jaugeon et al., 2025). Strong genetic structure has been detected at genomic markers among sites from Scotland to Portugal (Guzinski et al., 2020). Within‐population genetic diversity was lowest for the southern populations (Spain and Portugal) and the isolated island population on Helgoland, German Bight, and highest in Spitsbergen for both single nucleotide polymorphism (SNP) and expressed‐sequence‐tag (EST)‐derived microsatellites (Guzinski et al., 2020). This pattern was in agreement with the hypothesis of a high‐latitude refugia that has persisted through glaciation cycles (Jaugeon et al., 2025). A survey of 21 sites along the Norwegian coastline using nine microsatellite loci showed a relatively uniform genetic structure along 1000 km (Ribeiro et al., 2022). By contrast, fine‐scale genetic structure and low within‐population genetic diversity have been observed using microsatellite markers along the Maine region, United States (Breton et al., 2018).

Despite its importance as a foundation species and for the seaweed farming industry, baseline levels of genetic and genomic diversity have never been recorded in *Saccharina latissima* from the Estuary and Gulf of St. Lawrence. A recent study of *Zostera marina* revealed significant levels of genetic structuring in the Estuary and Gulf of St. Lawrence (Treillefort, 2023). By contrast, the few studies that have examined genetic structure in animals that disperse more actively have noted very weak structuring (Ferchaud et al., 2022). Populations of northern shrimp, *Pandalus borealis*, from the Estuary did not differ genetically from those from the northern Gulf (Bourret et al., 2024). Genetic differentiation was also very low among larger sampling regions: Estuary and northern St. Lawrence Gulf, Scotian Shelf, Newfoundland and Labrador, and the Flemish Cap at neutral SNP (Bourret et al., 2024).

**FIGURE 1:**
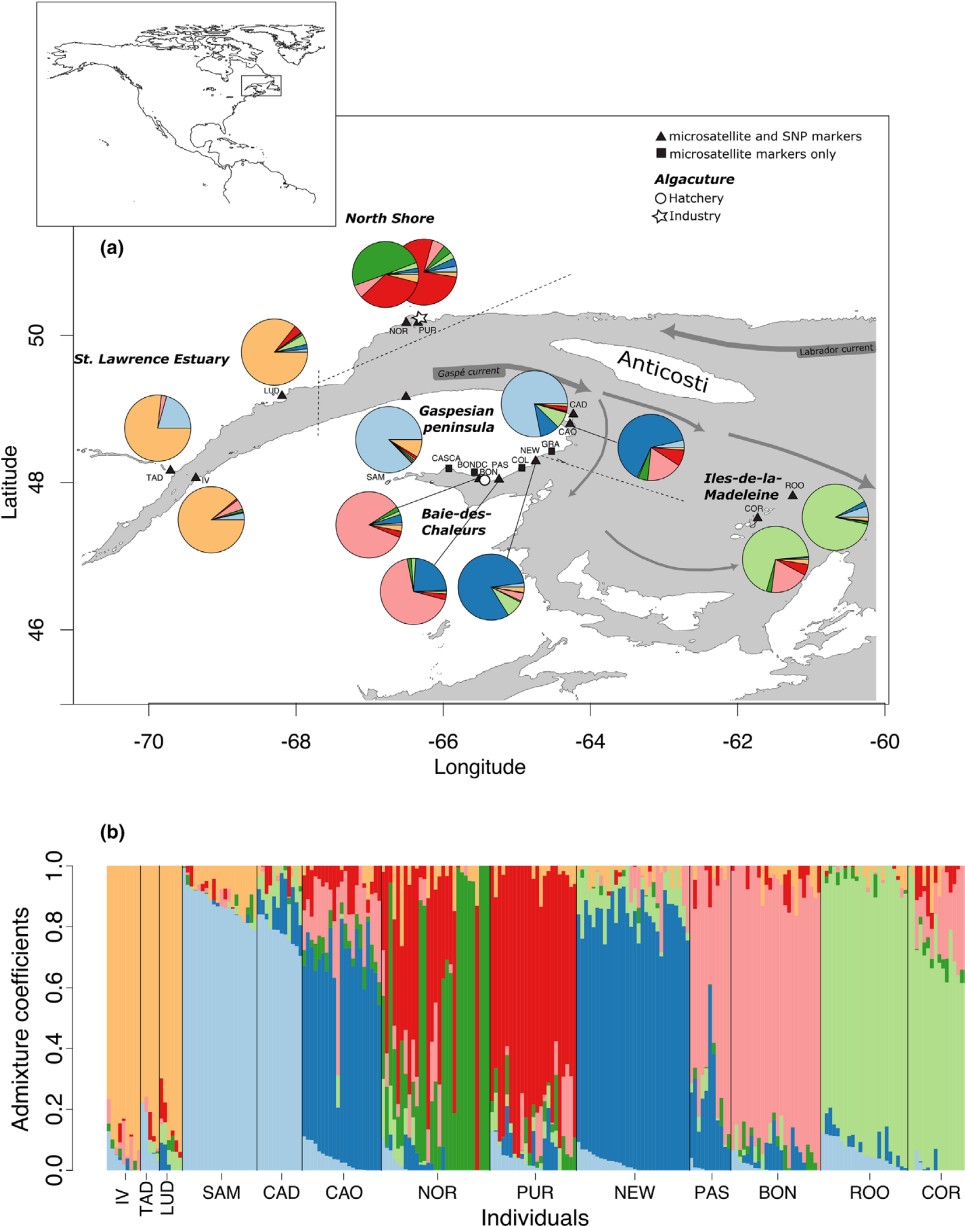
(a) Map of the sampling sites for *Saccharina latissima* in the Estuary and Gulf of St. Lawrence, Quebec, Canada. The source population site (BON) is represented with a circle, and the industry farm (PUR) with a star. Pie charts correspond to the relative proportions of the seven putative ancestral populations inferred by Bayesian clustering of the genetic diversity at SNP markers. (b) Graphical representation of ancestry estimates for 249 individuals, each represented by a vertical line with K segments colored proportionally to their belonging to a genetic cluster (*k* = 7). The percent ancestry is plotted on the *y*‐axis.

This study aimed to characterize genetic diversity and assess the genetic structure of wild populations of *Saccharina latissima* from the St. Lawrence seaway using microsatellite and genomic markers. As this species does not disperse actively, we expected currents to drive much of the genetic structure at neutral SNP (Jaugeon et al., 2025). We expected that sites located on the North Shore would be differentiated from other sites because of the presence of the Anticosti gyre (Figure 1). The Gaspé Current outflows from the St. Lawrence River, which moves around the Gaspé Peninsula and along the southern shore of the Gulf of St. Lawrence (Savenkoff et al., 1996). Therefore, sites in the Estuary and the Gaspé coast may form another genetic cluster. Finally, sites from the southern Gaspé coast and the southern Gulf should be genetically distinct due to the downward movement of the Gaspé Current. As individuals of *S. latissima* may dislodge from lines during their cultivation, there may be genetic contamination by individuals grown from the Baie‐des‐Chaleurs in the North Shore population. Assuming that sugar kelps from these two areas are genetically distinct, genetic contamination would result in sporophytes from areas near the culture sites being more genetically similar to those from the Baie‐des‐Chaleurs sites than to populations from their own region. More subtle genetic contamination may be revealed through higher observed heterozygosity levels at the farm site. Information on this key species can help create appropriate recommendations for managing aquaculture and preserving biodiversity in the St. Lawrence Gulf and Estuary.

## MATERIALS AND METHODS

### Microsatellite markers

#### Sampling

Adult sporophytes of *Saccharina latissima* (*n* = 480) were sampled throughout the Estuary and Gulf of St. Lawrence in 2018 and 2019. Thirty individuals were collected at each site in the Estuary: Tadoussac (TAD), Ile verte (IV), Baie St‐Ludger (LUD); in the Gulf along the North Shore: Ile Grosse Boule (Purmer algaculture site: PUR), Sept‐Iles (NOR); along the Gaspé Peninsula: Sainte‐Anne‐des‐Monts (SAM), Cap Desrosiers (CAD), Cap‐aux‐Os (CAO), Grande‐Rivière (GRA); in the Baie‐des‐Chaleurs: Newport (NEW), Colbourne (COL), Paspébiac (PAS), sporophytes from Bonaventure on growing lines (BONDC), Bonaventure (BON), and Cascapedia (CASCA); and around the Iles‐de‐la‐Madeleine: Rochers‐aux‐Oiseaux (ROO) and Cormorandière (COR; Figure 1; Table 1). Two small pieces of tissue (1 cm^2^) were cut from the basal blades of individual sporophytes and placed in silica gels until DNA extraction. The site PUR was located in the vicinity of the growing lines and BONDC consisted of sporophytes on the growing lines.

**TABLE 1:** Sampling site and their GPS coordinates of *Saccharina latissima* individuals in the St. Lawrence Estuary and Gulf.

Site	Latitude	Longitude
Tadoussac (TAD)	48.133119	−69.705842
Ile verte (IV)	48.034772	−69.340821
Baie St‐Ludger (LUD)	49.161609	−68.173798
Ste‐Anne‐des‐Monts (SAM)	49.130887	−66.500031
Pointe‐Noire (NOR)	50.163282	−66.467896
Ile Grosse Boule (PUR)	50.151276	−66.325630
Cap‐des‐Rosiers (CAD)	48.859972	−64.190300
Cap‐aux‐Os (CAO)	48.840380	−64.331696
Grande‐Rivière (GRA)	48.392669	−64.501934
Newport (NEW)	48.263541	−64.735703
Colborne (COL)	48.181379	−64.895210
Paspébiac (PAS)	48.017946	−65.265587
Bonaventure (BONDC)	48.031149	−65.479357
Bonaventure(BON)	48.041124	−65.491133
Cascapédia (CASCA)	48.175107	−65.918175
Cormorandière (COR)	47.496607	−61.723938
Rocher‐aux‐Oiseaux (ROO)	47.806514	−61.232300

*Note*: BON is the source site and PUR is the farm site.

#### DNA extractions

Total genomic DNA was extracted from 5 to 10 mg of dry tissue using the Nucleospin 96 plant kit (Macherey‐Nagel, Germany). The extractions were performed according to the manufacturer's instructions, except that samples were left in the lysis buffer at room temperature for 1 h rather than being heated to 65°C for 30 min. The extracted DNA was eluted in 200 μL of the supplied elution buffer and one additional wash with buffer PW1 and one additional wash with buffer PW2 to more effectively remove polymerase chain reaction (PCR) inhibitors and polysaccharides.

#### Microsatellite loci amplification

Overall, 44 microsatellite loci for *Saccharina latissima* were amplified. Twelve of these loci were previously described in Paulino et al. (2016), and 32 Expressed Sequenced Tag (EST)‐derived microsatellite loci wereselected from the study of Guzinsky. For the multiplexes with the 12 loci characterized by Paulino et al. (2016), PCR mixes were prepared following their instructions except for deoxyribonucleotides (dNTPs, which were adjusted to 150 μM) and the Taq polymerase (adjusted to 0.5 U). For the multiplexe from the ESTs, amplifications were carried out in 20‐μL reaction volumes with each reaction comprising 2 μL of DNA template diluted 1:50 (to help reduce PCR inhibitors), 2 mM of MgCl_2_, 1 × GoTaq® Flexi PCR buffer, 150 μM of dNTP, 400 nM of forward primer, 400 nM of reverse primer and 0.5 U of GoTaq® Flexi (Promega Corporation, Madison, Wisconsin, United States). All PCR amplifications were performed using a T100TM Thermal Cycler (Bio‐Rad Laboratories, Inc., Hercules, California, United States), and parameters used were described in Paulino et al. (2016) and Guzinski et al. (2016) for the EST multiplexes. Next, 2 μL of PCR product was added to 10 μL of loading buffer made up of 0.5 μL of the SM594 size standard (Mauger et al., 2012) and 9.5 μL of Hi‐Di formamide, denatured at 95°C for 3 min, and run in an ABI 3130 XL capillary sequencer (Applied Biosystems, United States). Genotypes were scored manually in Genemapper version 4.0 (Applied Biosystems) and verified by two readers.

#### Genomic markers

A total of 282 adult sporophytes of *Saccharina latissima* selected from 13 locations were sent to Diversity Arrays Technology (DArT) at the University of Canberra (Australia). Three sites were in the St. Lawrence Estuary (TAD, IV, and LUD), two were along the North Shore (PUR and NOR), three were along the Gaspé Peninsula (SAM, CAD, and CAO), three were in the Baie‐des‐Chaleurs (NEW, PAS, and BON), and two were around the Iles‐de‐la‐Madeleine (ROO and COR). Libraries were constructed according to Kilian et al. (2012). DArTseq is a proprietary genome complexity reduction‐based sequencing technology that differs from other methods by its ability to select the predominantly active—low copy sequence—areas in a genome, which are the ones containing the most useful information. At the same time, DArTseq masks the lesser value, repetitive sequences. It does this through the application of a combination of restriction enzymes to fragment DNA samples in a highly reproducible manner. DArTseq complexity reduction method was used through digestion of genomic DNA with two restriction enzymes (*Pst*I and *Mse*I) and ligation of barcode adapters followed by PCR amplification of adapter‐ligated fragments. Libraries were sequenced using single‐read sequencing runs for 77 bases by Hiseq2500. In the primary pipeline the fastq files were first processed to filter away poor quality sequences, applying more stringent selection criteria to the barcode region compared to the rest of the sequence (minimum Phred pass score = 30 and minimum pass percentage = 75). Approximately 2,500,000 sequences per barcode/sample were identified and used in marker calling. Finally, identical sequences were collapsed into “fastqcoll files.” The fastqcoll files were “groomed” using DArT PL's proprietary algorithm, which corrects low‐quality bases from singleton tag into a correct base using collapsed tags with multiple members as a template. The “groomed” fastqcoll files were used in the secondary pipeline for DArT PL's proprietary SNP and SilicoDArT (presence/absence of restriction fragments in representation) calling algorithms (DArTsoft14). Thirty replicates were produced to assess the reproducibility of the sequences and average reproducibility was 99%. Markers were aligned to the *S. japonica* (SJ6l.1) genome.

### Data analysis

#### Microsatellite markers

The presence of sequencing errors like stuttering bands, allelic dropouts, and null alleles was verified with the MICRO‐CHECKER software version 2.2.3 (Van Oosterhout et al., 2004). Monomorphic loci, loci with more than 30% of missing data, and loci with missing data for all individuals for a site were removed from the database. All individuals with more than three missing data points per loci were also removed from the database. After selection, 22 loci were retained for 308 individuals. All locations were kept for analyses except CAO, since it contained too much missing data. The number of individuals per site ranged from nine to 28. Allelic richness was estimated in FSTAT, version 2.9.4 (Goudet, 2005a).

#### Genomic markers

Initially, we received 68,971 single‐row SNP markers from DArT Pty Ltd. These DArT loci were then filtered using the dartR library (Gruber et al., 2018) in Rstudio (version 4.2; Rstudio, 2015) to obtain only informative loci. Four filters were used. We removed (1) all the monomorphic loci (27,525 remaining SNPs in 282 individuals), (2) the loci with more than 0.5% of missing data (20,131 remaining SNPs in 282 individuals), (3) the duplicate loci (18,623 remaining SNPs in 282 individuals), and (4) the individuals with more than 20% of missing data (33 individuals). After recalculating the loci call rate, an additional 11,771 loci were removed, resulting in a total of 6582 SNPs remaining in 249 individuals. The number of individuals ranged from nine to 30, except for at TAD, which had only two individuals and was not considered for diversity indices estimations. Genetic diversity indices and population differentiation analyses were estimated only for sampling sites with a minimum sample size of nine individuals.

#### Both marker types

Genetic diversity indices were estimated using the hierfstat package (Goudet, 2005b). Inbreeding coefficients (*F*_IS_) were calculated together with these indices, and the significance was calculated with 1000 iteration bootstraps. Genetic differentiation was estimated with the hierfstat package (Gruber et al., 2018) using fixation index *F*_ST_ (Weir & Cockerham, 1984), and its statistical significance was tested by performing 1000 bootstraps.

A principal component analysis (PCA) was performed to allow a visual interpretation of the genetic structure of the data using the DartR package version 2.9.7 (Gruber et al., 2018). The analyses of isolation by distance were tested using the in‐water non‐Euclidean paths as the distance between sites. These data were retrieved using the marmap package version 1.0.10 (Pante & Simon‐Bouhet, 2013). Individual ancestry coefficients for both microsatellites and the SNP data were estimated using the sparse non‐negative matrix factorization (sNMF) method (Frichot et al., 2014) as implemented in the R package LEA (Frichot & François, 2015). The sNMF method is robust against deviations from the Hardy–Weinberg equilibrium and does not rely on any a priori population genetic assumptions (Frichot et al., 2014). This method offers comparable performance to Structure and ADMIXTURE while providing significantly faster run times for genomic datasets. The number of putative clusters (*K*) ranged between 1 and the number of sampling sites (depending on the marker), and for each *K*, 100 repetitions with 99 maximum iterations were carried out. Ten percent of the genotypes were masked at each run to compute the cross‐entropy criterion. The *K* with the smallest cross‐entropy value and/or from which this parameter stabilized was selected. Analyses of molecular variance (AMOVA) were performed to examine the distribution of genetic variation within and among populations using the poppr R package (Kamvar et al., 2015). This analysis, analogous to an analysis of variance (ANOVA) analysis, partitions genetic diversity at multiple hierarchical levels, such as within individuals, among individuals within populations, and among populations themselves, and allows for the identification of genetic population structure. Group discrimination was based on PCA results on SNP markers—Group 1: IV, LUD, SAM, and CAD; Group 2: NOR and PUR; Group 3: NEW, BON, PAS, and CAO; and Group 4: Iles‐de‐la‐Madeleine (ROO and COR)—and applied to both microsatellite and SNP data.

## RESULTS

### Genetic diversity

**FIGURE 2:**
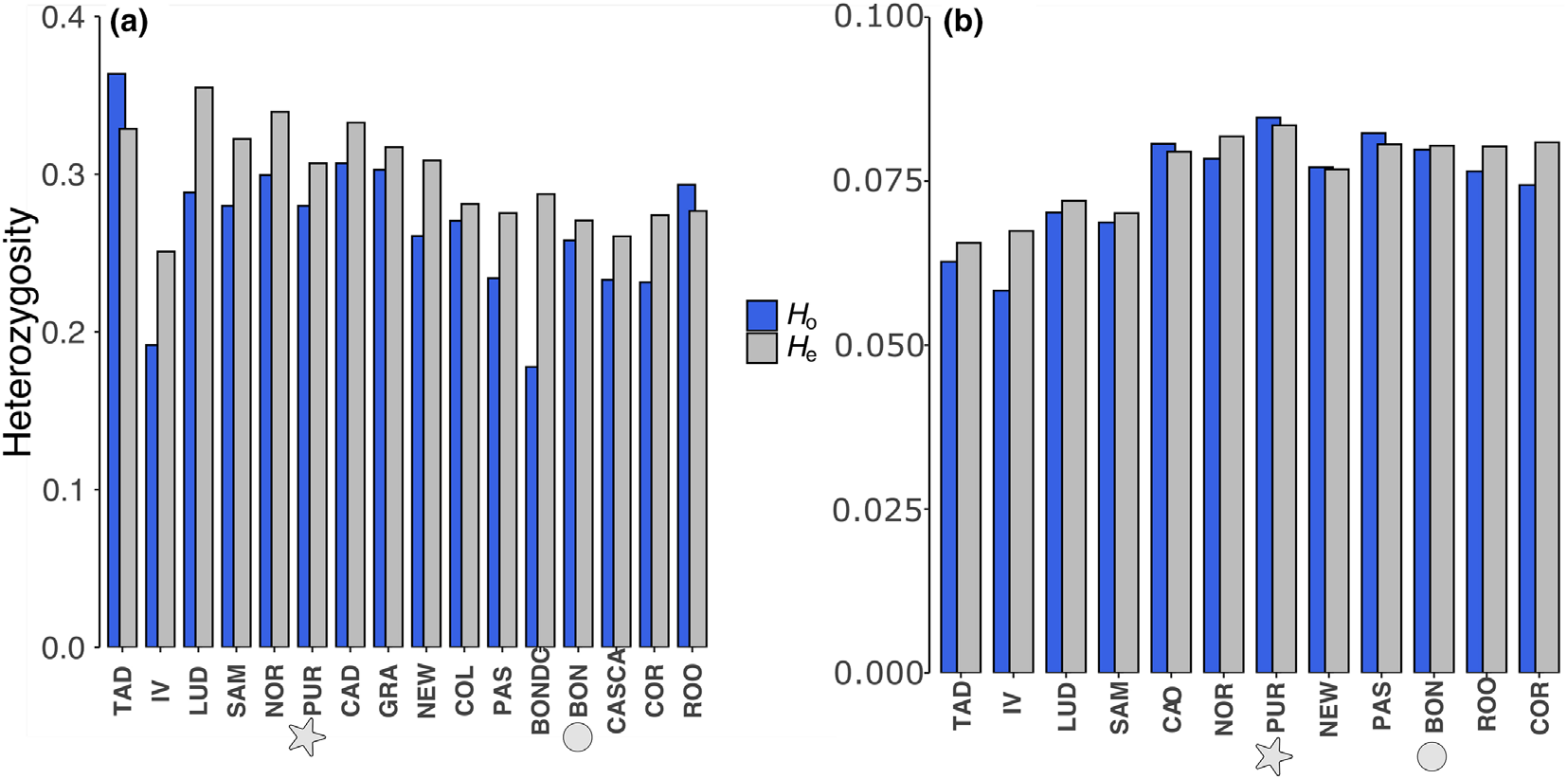
Heterozygosities (observed and expected) estimated from (a) microsatellite markers and (b) SNP and presented for each sampling site.

Genetic diversity indices calculated with microsatellite markers were relatively low, with expected heterozygosity (*H*e) values ranging from 0.249 to 0.339 and a mean number of alleles of 2.8 on average per site (Table 2, Figure 2a). Moreover, more than half of the sites had positive and significant *F*_IS_ values. Only five sites were in Hardy–Weinberg equilibrium; the others showed heterozygote deficiency. Genetic diversity indices using SNP indices were 10× lower, with *H*e ranging from 0.0656 (IV) to 0.083 (PUR; Table 2, Figure 2b). Populations from the Estuary and Gaspé Peninsula (CAD, SAM, IV, LUD) had lower heterozygosity than the rest of the sampling sites. Seven sites had positive *F*_IS_ values; five of them also showed positive values at microsatellite markers. Sites from the Estuary had lower heterozygosity values than those from the Gulf at SNP but not at microsatellite markers. The farm site (PUR) had lower allelic richness and heterozygosity than the wild site in the vicinity (NOR).

**TABLE 2:** Genetic diversity indices for microsatellite and SNP methods: Sample size (*n*), expected heterozygosity (*H*e), mean allelic richness (AR), inbreeding coefficient values (*F*_IS_).

Site	Microsatellites				SNPs		
	*n*	*He*	AR	*F*_IS_	*n*	*He*	*F*_IS_
TAD	14	0.330	1.68	−0.106	–	–	–
IV	15	0.249	1.51	0.236*	9	0.0656	0.0396*
LUD	9	0.336	1.66	0.188	9	0.0671	0.1040*
SAM	22	0.321	1.66	0.131*	20	0.0719	0.0200*
NOR	22	0.339	1.71	0.118*	29	0.0890	0.0256*
PUR	20	0.306	1.64	0.088*	23	0.0833	−0.0063
CAD	19	0.332	1.69	0.077*	12	0.0701	0.0158*
CAO	–	–	–	–	21	0.0795	−0.0076
GRA	15	0.317	1.66	0.045	–	–	–
NEW	21	0.308	1.66	0.156*	30	0.0764	0.005
COL	23	0.280	1.58	0.038	–	–	–
PAS	23	0.274	1.57	0.150*	11	0.081	−0.0141
BONDC	13	0.281	1.6	0.381*	–	–	–
BON	23	0.270	1.58	0.047	24	0.0804	0.0102
CASCA	22	0.260	1.53	0.105*	–	–	–
COR	21	0.273	1.56	0.155*	13	0.0809	0.0611*
ROO	26	0.277	1.58	−0.060	25	0.0803	0.0368*

*Note*: Values in bold with an asterisk are significant. BON is the source site and PUR is the farm site.

### Genetic structure

**FIGURE 3:**
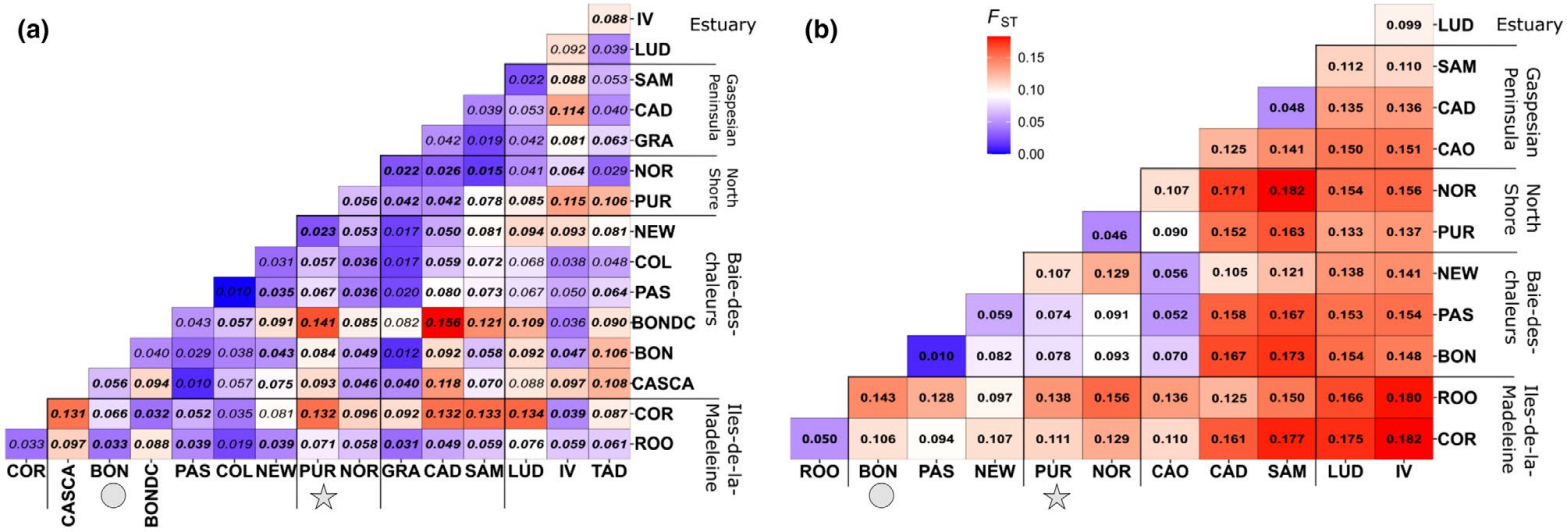
Matrix of pairwise *F*_ST_ estimated from (a) microsatellite and (b) SNP markers. Values in bold represent significant values. Significance was tested using bootstrapping (*N* = 10,000) and significant values are presented in bold. Pairwise comparisons most relevant are PUR‐BON, NOR‐BON, and PUR‐NOR.

**FIGURE 4:**
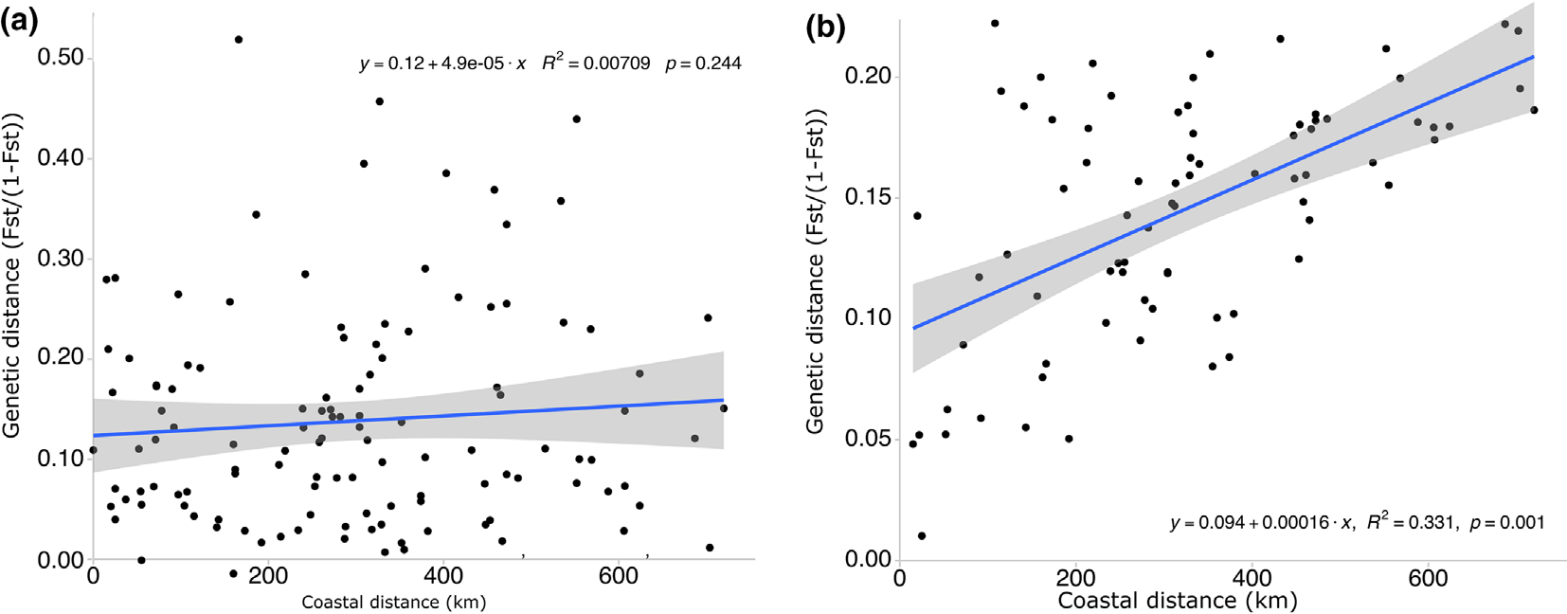
Mantel test plot performed using (a) microsatellites, and (b) SNP markers displaying the correlation between genetic and coastal distances among sugar kelp populations. Correlation coefficient *r* and *p*‐value are shown.

**FIGURE 5:**
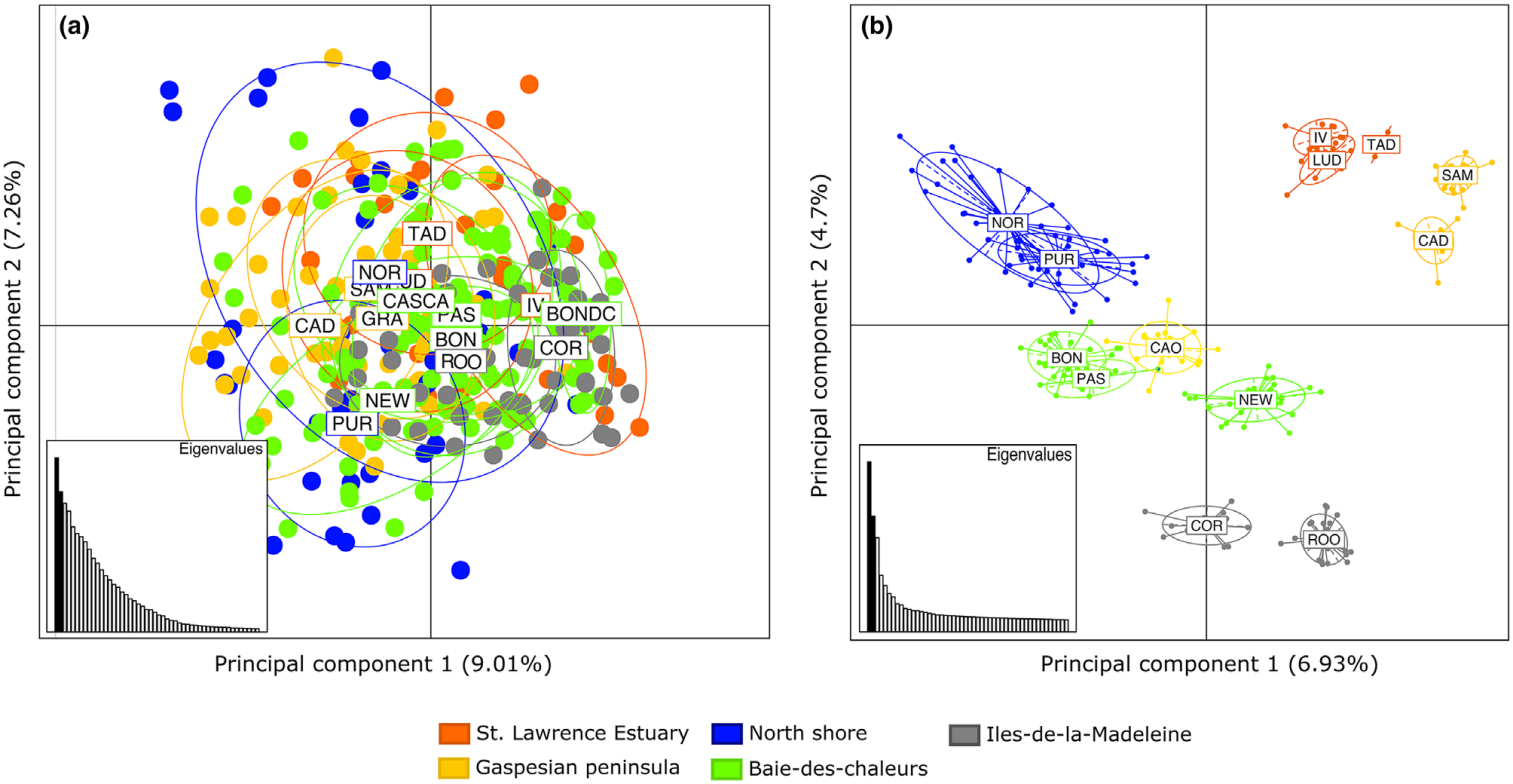
Principal Component Analysis (PCA) based on (a) microsatellite and (b) SNP markers from individuals of *Saccharina latissima* (*n* = 228) collected in the Estuary and Gulf of St. Lawrence. Each point represents an individual, and each color corresponds to one site. TAD = Tadoussac, IV=Ile verte, LUD=Baie de St‐Ludger, PUR = Purmer algaculture site, NOR = Sept‐Iles, SAM = Sainte‐Anne‐des‐Monts, CAD=Cap Desrosiers, CAO=Cap‐aux‐Os, GRA = Grande‐Rivière, NEW=Newport (NEW), COL = Colbourne, PAS=Paspébiac, BONDC = growing lines in Bonaventure, BON = Bonaventure, CASCA = Cascapedia, ROO = Rochers‐aux‐Oiseaux, COR = Cormorandière. The percentage of variation explained by both axes is shown.

The matrix of pairwise *F*_ST_ (Figure 3a) values revealed low genetic differentiation among the 16 locations using microsatellite markers. Significant *F*_ST_ values were observed between the Iles‐de‐la‐Madeleine location (ROO) and most of the other sites and between the Sept‐Iles site and many locations. *Saccharina latissima* from Bonaventure (BON) used for algal culture showed small but significant genetic differentiation with individuals collected in NOR (located 1 km from the cultivation line on the North Shore). All pairwise *F*_ST_ obtained from SNPs were significant, with the highest values between the Iles‐de‐la‐Madeleine and the Estuary (from 0.18 to 0.12) and the lowest values among sites within the Baie‐des‐Chaleurs (from 0.01 to 0.08; Figure 3b). Individuals from PUR (aquaculture site) showed little genetic differentiation from NOR individuals located 1 km from that site and were genetically differentiated from the Baie‐des‐Chaleurs site (BON), where meiospores were collected from 2014 to 2018. Mantel tests revealed no significant isolation by distance at microsatellites (Figure 4a) but a significant relationship at SNPs (Figure 4b). The PCAs from microsatellite markers revealed the presence of a single group composed of all 16 sites (Figure 5a). By contrast, the PCA with SNP markers revealed significant structure, with Estuary (TAD, IV, LUD) and Gaspé Peninsula sites (SAM and CAD) forming the first group and North Shore sites (NOR and PUR) on the first axis (6.93% of total genetic variance) forming a second group. The Baie‐des‐Chaleurs sites (BON, PAS, and NEW), CAO, and the Iles‐de‐la‐Madeleine sites (ROO and COR) separated along the second principal component (4.71%; Figure 5b) and formed the third group.

Population structuration analyses determined that one ancestral population was the most likely scenario with microsatellite markers, with the minimum cross‐entropy recorded at *K* = 1. When using the SNP markers, the most probable number of ancestral populations was seven, after which the cross‐entropy criterion reached a plateau (Figure 1). The estuarian sites (IV, TAD, and LUD) all shared a common major ancestral cluster, the Gaspesian sites (SAM and CAD) shared another one, and the sites from Iles‐de‐la‐Madeleine (ROO and COR) formed a third cluster (Figure 1). The CAO site, separated from CAD on the Gaspé peninsula by narrow land, formed a group with NEW. The sites located deeper in the Baie‐des‐Chaleurs (PAS and BON) formed another cluster. The sites from the north shore (NOR and PUR) were separated into distinct clusters.

*F* analyses explained only 0.71% of the variation at microsatellite loci—this percentage was not significant—whereas the variance explained by the four groups at SNP markers accounted for more than 7% of the total variation and was significant (*F*_SR_ = 0.045, *p* = 0.01; Table 3).

**TABLE 3:** Analysis of molecular variance (AMOVA) using four groups based on group discrimination revealed by PCA on SNP markers: Group 1: IV, LUD, SAM, and CAD; Group 2: NOR and PUR; Group 3: NEW, BON, PAS, and CAO; and Group 4: Iles‐de‐la‐madeleine (ROO and COR).

Markers	Source of variation	*df*	Sum of squares	Variance components	Percentage of variation	*p*‐value
Microsatellites	Among groups	3	66.638	22.21	0.71	0.16
	Among populations within groups	12	184.75	15.39	4.54	**0.01**
	Among individuals within populations	292	1687.15	5.78	7.98	**0.01**
	Within individuals	308	1503.01	4.88	86.76	**0.01**
	Total	615	1625.60	5.60	100	
SNPs	Among groups	3	18,731.33	6243.7762	7.30	**0.01**
	Among populations within groups	8	13,182.29	1647.7862	6.04	**0.01**
	Among individuals within populations	214	10,3574.25	483.9918	1.87	0.07
	Within individuals	226	104,755.91	463.5217	84.77	**0.01**
	Total	451	240,243.77	532.6913	100	

*Note*: BON is the source site and PUR is the farm site. Significant *p* values less than 0.05 are indicated in bold.

## DISCUSSION

This study examined patterns of genetic diversity and structure in wild populations of *Saccharina latissima* from the St. Lawrence Estuary and Gulf. Results showed low genetic diversity in *S. latissima* from the St. Lawrence seaway, with half of the sites showing high *F*_IS_ indices and a low number of effective alleles per locus. Observed low levels of genetic diversity may be due to bottlenecks following postglacial colonization in the St. Lawrence River. A similar study in Maine, United States, observed higher levels of allelic richness (3.6 and 4.2) with only six microsatellite markers and no inbreeding (Breton et al., 2018). A worldwide survey using 12 microsatellite loci reported that the Eastern Atlantic (Europe) populations were more genetically diversified than the Western Atlantic (US and Canadian Coasts) and the Pacific populations (Neiva et al., 2018). Heterozygosity levels were lower in NW Atlantic *S. latissima* populations, and several sites had high inbreeding levels (Neiva et al., 2018). Three of our sites were sampled in this Neiva et al. (2018) study (NOR, NEW, and BON) and had an average allelic richness of 3.9, which was similar to other populations from the NW Atlantic but higher than our values (around 3). In contrast to our present results comparing the same sites, Neiva et al. (2018) did not detect any inbreeding at our three locations but did at several other NW sites including the Canadian Maritimes. Allelic richness was twice as high in NE Atlantic populations compared to our results for the same set of microsatellite loci (Guzinski et al., 2016). Higher levels of genetic diversity in the NE than in the NW populations have also been observed in *Laminaria digitata* (Neiva et al., 2020). Guzinski et al. (2020) observed higher levels of genetic diversity at SNPs (from ddradseq) in the northernmost localities of Norway and Scotland (*H*o of 0.05) than in more southern populations located in Portugal (*H*o of 0.030). Among studies, comparisons of genetic diversity indices are more difficult with SNP than with microsatellites, since the type of sequencing can differ as can the sequencing coverage as well as the various filtrations on the datasets.

Recurrent bottlenecks following Pleistocene glaciation may have affected the genetic diversity of sugar kelp in the St. Lawrence. During this period, 25,000–18,000 years ago, the ice sheet covered North America, from the Arctic to approximately New York, and the St‐Lawrence was wholly sealed in ice (Maggs et al., 2008). After this period, many species, such as *Saccharina latissima*, recolonized ice‐free areas. It has been assumed that populations of the NW Atlantic were composed of individuals that migrated across the Atlantic Ocean from Europe following the last glaciation (Lee, 1973; Luttikhuizen et al., 2018; Taylor, 1957). Neiva et al. (2018) suggested that NW and NE lineages evolved in the Atlantic and split into two groups prior to the late glacial maximum. The consequences of glaciations were harsher in the NW Atlantic due to the paucity of rocky shores, resulting in lower genetic diversity in members of this phylogroup. Few studies have examined recolonization patterns in species from the St. Lawrence. The anadromous rainbow smelt (*Osmerus mordax*) recolonized the St. Lawrence Estuary from two refugia (Bernatchez, 1997). The Mysid *Neomysis americana* includes two different lineages, one that would have colonized the St. Lawrence via a southern refuge around Georges Bank with a migration route via the Champlain Sea, and another one with an origin east of the Grand Banks, Newfoundland (Cortial et al., 2019). Few studies have compared genetic diversity among the Estuary and the Gulf of St. Lawrence in species that do not actively disperse. Our results showed low levels of genetic diversity, suggesting recolonization from a small number of individuals and a single refugium. Additional studies examining mitochondrial genomes would be needed for confirmation. Environmental factors may also affect genetic diversity levels. Populations from the more brackish environment (lower Estuary) were less genetically diverse than those from the Gulf at genomic markers. A similar result was observed for *Zostera marina* populations from the same area and sequenced with the same technique (Dartseq; Treillefort, 2023). Nielsen et al. (2016) observed that brackish populations of *S. latissima* in Denmark were less genetically diverse than marine populations and likely more vulnerable to climate change due to their genetic isolation.

A very weak genetic structure was revealed for the 22 microsatellite markers in comparison with SNPs. The use of genomic markers allowed an increased resolution and the detection of restriction to gene flow at major barriers in the St. Lawrence Seaway. Spatial analysis of genetic distance using SNPs supported the influence of isolation by distance (IBD) on population differentiation across the whole region. The dataset also supported the presence of important hierarchical structuring. The Gaspé Current flows along the south shore of the Estuary and along the Gaspé Peninsula, thus explaining the first and second cluster comprising individuals from the lower Estuary (IV, TAD, and LUD) and the Gaspé Peninsula (SAM and CAD). The isolation of North Shore populations (NOR and the algal culture site, PUR) can be explained by the presence of a gyre near Anticosti Island (Archambault et al., 2017). These two sites were observed to be distinct from one another despite the low *F*_ST_ values between them. Additional samples at sites located to the east of this location would help in the understanding of the genetic divergence between these sites. The populations (BON and PAS) that are located to the west of the Baie‐des‐Chaleurs clustered together, whereas NEW and CAO, occupying the eastern part of that area, formed a distinct cluster. The Gaspé Current flowing from the Gaspé Peninsula to the Iles‐de‐la‐Madeleine (Savenkoff et al., 1996) likely prevents mixing between these sites and the sites from the Gaspé Peninsula. The Iles‐de‐la‐Madeleine (ROO and COR) are further isolated due to distances from the rest of the sites. Oceanographic processes appear to be more important drivers than geographic distance for finer spatial resolution in populations of *Laminaria digitata* (Fouqueau et al., 2024). In Maine, *Saccharina latissima* showed genetic differentiation within a few kilometers: Four groups were detected with microsatellites along only 210 km of shoreline (Breton et al., 2018). In the eastern United States, the Gulf of Maine and southern New England populations were separated by Cape Cod, a peninsula that appears to be a biogeographic barrier to the gene flow in this area (Mao et al., 2020). In comparison, the Gaspesian Peninsula could be a barrier to the gene flow separating the Estuary, the North Shore, and the Gaspesian coast from the Baie‐des‐Chaleurs.

The use of locally sourced seeding material has been advocated to reduce the risk of genetic pollution and depression from introduced non‐local strains (Yarish et al., 2017). Low levels of introgression have been reported in naturalized populations of *Undaria pinnatifida* from nearby farm sites in France (Grulois et al., 2011). A recent study comparing 24 wild and three farmed *Saccharina latissima* populations at 21 microsatellite loci revealed no significant genetic differentiation between them, reflecting the current cultivation practices of using local meiospores in Brittany, France (Jaugeon et al., 2025). In China, few signs of gene flow from farm to wild have been reported in U. *pinnatifida* (Li et al., 2020). Macroalgae farming has been in operation at very small scales since 2006 in the St. Lawrence. Our study revealed that the wild population of the Baie‐des‐Chaleurs (BON), where the broodstock is collected, is genetically distinct from the wild population of Sept‐Iles. The North Shore population where the aquaculture has recently been in operation (PUR) did not differ genetically from the wild population (NOR) located 1 km away and did not have higher levels of genetic diversity as would have been expected if there was admixture. This suggests an absence of genetic contamination from the seed broodstock. As sea farming has not been practiced intensively, our results are not surprising. As genetic diversity appears limited in our populations and genetic structure is high, it would be important to take this into consideration for selection programs and if farming activities intensify. The introduction of genetic diversity through the crossing of divergent populations of *S. latissima* has been demonstrated to result in heterosis, leading to increased yield (Cohen et al., 2025). The recent report of sporeless sporophyte production could alleviate the problem of genetic contamination and enable the production of desirable traits in cultivated *S. latissima* (Vissers et al., 2024). However, such a strategy raises the issue of farmers' dependence on seed producers. To promote more independence from seed producers, it will be important to maintain/explore alternative options such as local strains adapted to their specific populations.

## AUTHOR CONTRIBUTIONS

**Marie Treillefort:** Formal analysis (equal); methodology (equal); writing – original draft (equal). **Sabrina Le Cam:** Formal analysis (equal); software (lead); writing – original draft (equal); writing – review and editing (equal). **Myriam Valero:** Funding acquisition (equal); supervision (supporting); writing – review and editing (equal). **Stéphane Mauger:** Methodology (equal); supervision (supporting); writing – review and editing (supporting). **Paolo Ruggeri:** Software (equal); supervision (supporting); writing – review and editing (supporting). **Flora Salvo:** Funding acquisition (equal); supervision (equal); writing – review and editing (equal). **Isabelle Gendron‐Lemieux:** Funding acquisition (supporting); writing – review and editing (supporting). **Tamara Provencher:** Funding acquisition (supporting); project administration (supporting); writing – review and editing (supporting). **Rénald Belley:** Funding acquisition (supporting); writing – review and editing (supporting). **France Dufresne:** Conceptualization (equal); formal analysis; funding acquisition; investigation (equal); methodology; project administration; supervision (lead); writing – original draft; writing – review and editing.
